# The microbial damage and host response framework: lesson learned from pathogenic survival trajectories and immunoinflammatory responses of *Talaromyces marneffei* infection

**DOI:** 10.3389/fimmu.2024.1448729

**Published:** 2024-08-12

**Authors:** Kritsada Pruksaphon, Artid Amsri, Juthatip Jeenkeawpieam, Patcharin Thammasit, Joshua D. Nosanchuk, Sirida Youngchim

**Affiliations:** ^1^ Department of Medical Technology, School of Allied Health Sciences, Walailak University, Nakhon Si Thammarat, Thailand; ^2^ Center of Excellence Research for Melioidosis and Microorganisms (CERMM), Walailak University, Nakhon Si Thammarat, Thailand; ^3^ Office of Research Administration, Chiang Mai University, Chiang Mai, Thailand; ^4^ Department of Microbiology, Faculty of Medicine, Chiang Mai University, Chiang Mai, Thailand; ^5^ Akkhraratchakumari Veterinary College, Walailak University, Nakhon Si Thammarat, Thailand; ^6^ One Health Research Center, Walailak University, Nakhon Si Thammarat, Thailand; ^7^ Department of Medicine (Division of Infectious Diseases) and Department of Microbiology and Immunology, Albert Einstein College of Medicine, New York, NY, United States

**Keywords:** *Talaromyces marneffei*, immunopathogenesis, damage-response framework (DRF), latency, immune paradox, IRIS, HLH

## Abstract

The adverse outcomes of fungal infection in mammalian hosts depend on the complex interactions between the host immune system and pathogen virulence-associated traits. The main clinical problems arise when the host response is either too weak to effectively eliminate the pathogen or overly aggressive, resulting in host tissue damage rather than protection. This article will highlight current knowledge regarding the virulence attributions and mechanisms involved in the dual-sided role of the host immune system in the immunopathogenesis of the thermally dimorphic fungus *Talaromyces marneffei* through the lens of the damage response framework (DRF) of microbial pathogenesis model.

## 1 Introduction

“In many parasitic conditions, there is often limited pathology directly attributable to the parasite. Most host morbidity is related to the immunoinflammatory response of the host to the parasite.”

- Immunopathology of Parasitic Diseases: A Conceptual Approach by S. Michael Phillips and Edward G. Fox (1)

The aforementioned statement, while utilized to elucidate the pathogenesis of disease in the field of medical parasitology, bears relevance in the context of infection caused by diverse eukaryotic pathogens (e.g. protozoa, helminths and fungi) as there are certain similarities and observable phenomena. The thermally dimorphic fungus *Talaromyces marneffei* (previously known as *Penicillium marneffei*) is an understudied human fungal pathogen identified as a medium global health threat by the World Health Organization (2, 3). Especially due to its occurrence primarily in lower-middle-income countries, numerous questions on the pathobiology of *T. marneffei* persist. Nevertheless, we and others have demonstrated that the interplay between the host immune system and *T. marneffei* is critical to the outcome of infection.

In the field of medical microbiology, immunology and infectious diseases, the assessment of the degree and characteristic of harm inflicted upon a host organism by an opportunistic microorganism was historically predominantly contingent upon the virulence attributes of the pathogenic microorganism. In experimental models of infections, factors such as host genotype, fungal genotype, and other variables are deliberately controlled to ensure consistent disease outcomes. These experimental models offer significant advantages, allowing researchers to employ drastic measures like gene disruption to investigate the molecular mechanisms underlying particular pathological alterations. Nevertheless, it is crucial to acknowledge that the interpretations drawn from such data are context specific. Interventions that prove advantageous within a controlled experimental setting may not necessarily yield similar benefits under the diverse genetic and environmental conditions encountered in natural human or animal populations. Moreover, it is now understood that the extent of danger faced by the host during an infection is also contingent upon the immune condition of the host and is frequently influenced by the host responses. Consequently, the manifestation of disease itself is a multifaceted outcome, which may arise due to pathogenic microorganism-induced pathogenesis, host-induced pathogenesis, or a combination of both circumstances (4, 5). Consequently, in numerous interactions between pathogenic microorganism and healthy hosts, there exists a spectrum between damage caused primarily by the pathogenic microorganism and that primarily induced by the host, culminating in the emergence of disease only when the nature of the damage disrupts the host normal physiological function (homeostasis).

This complex paradigm has given rise to the concept of the Damage Response Framework for Microbial Pathogenesis (DRF for Microbial Pathogenesis) by Liise-anne Pirofski and Arturo Casadevall in 1999 (6), which delineates microbial pathogenesis as an outcome resulting from the interplay between a specific host and specific a microorganism. The DRF for Microbial Pathogenesis is founded on the fundamental tenet that true pathogens, commensals, and opportunistic microorganisms are not exclusive categories, but rather all microbial pathogenesis materializes when a microbe and a host interact, with the ultimate outcome being harm to the host. “Infection is common but disease is rare,” as articulated by Casadevall, appears to be the most straightforward and clear summary of the aforementioned concept. “Infection” occurs when pathogenic microorganisms successfully overcome the host’s defenses and adapt within the host, whereas “disease” is the consequence of the host’s immunological or physiological imbalances, resulting in the inability to maintain homeostasis due to infection, ultimately leading to variable levels of host damage and disease manifestations (7). Significantly, this adaptable conceptual framework encompasses the host and microorganism and outcomes influenced by numerous factors and surrounding environment as well as strong or weak immune responses to individual pathogenic microorganisms. Beyond pathogenic eukaryotes, the DRF theory has also been applied to pathogenic bacteria and viruses, particularly in explaining the pathogenesis and severeness of COVID-19 (8, 9).

In the field of *T. marneffei* infection, we have previously provided an overview of the pathogenic lifestyle and basic immune response to infection (10). Although the incidence of talaromycosis significantly increased with the onset of the HIV pandemic, the incidence has further increased since the availability of Highly Active Antiretroviral Therapy (HAART) era, raising interesting questions about the exact nature of pathogenesis in *T. marneffei* infections. Well-establishing knowledge asserts that this fungal species is an opportunistic pathogen; however, the extent of its pathogenicity and the role of the host immune system in controlling *T. marneffei* infections remains uncertain (11). Moreover, what is the effect on outcome with an insufficient or over robust host response to *T. marneffei* infection? This article aims to demonstrate that the pathogenesis of talaromycosis is intricately linked to the various degree spectrums of the host immune system and in regulating the progression to and severity of disease.

This review explores the impact of the pathogenic *T. marneffei* and host immune system on the outcome of infection, with a particular focus on granuloma formation, immune reconstitution inflammatory syndrome (IRIS) and macrophage activation syndrome (MAS) or secondary (acquired) hemophagocytic lymphohistiocytosis (HLH).

## 2 The conceptualization of the microbial damage and host response framework

The damage-response framework (DRF) model suggests that clinical disease can arise from either uncontrolled spread of the pathogen or an adverse (too weak or too strong) immune response by the host, both resulting in host damage. This concept has been extensively explained and studied in the case of cryptococcal infection (12, 13). The acknowledgment that both the host immune response and microbes have the potential to undergo changes naturally led to the inevitable realization that the outcome of their interaction could also undergo alterations. Considering that not all individuals exposed to a microbe fall ill and that the condition following infection can vary among patients or even within the same patient over time, Pirofski and Casadevall concluded that the fundamental aspect of host-microbe interaction, and the most pertinent factor for evaluating its outcome, should be host damage. This deduction led to the following principles of the DRF, which are considered to be incontrovertible: (**I**) microbial pathogenesis results from the interaction between a host and a microbe; (**II**) the outcome relevant to the host in this interaction is host damage; (**III**) host damage can arise from microbial (fungal) factors, host factors, or a combination of both. These principles enabled investigators to comprehensively address the roles of both the host and the microbe in the pathogenesis of infectious diseases (14).

**Figure 1:**
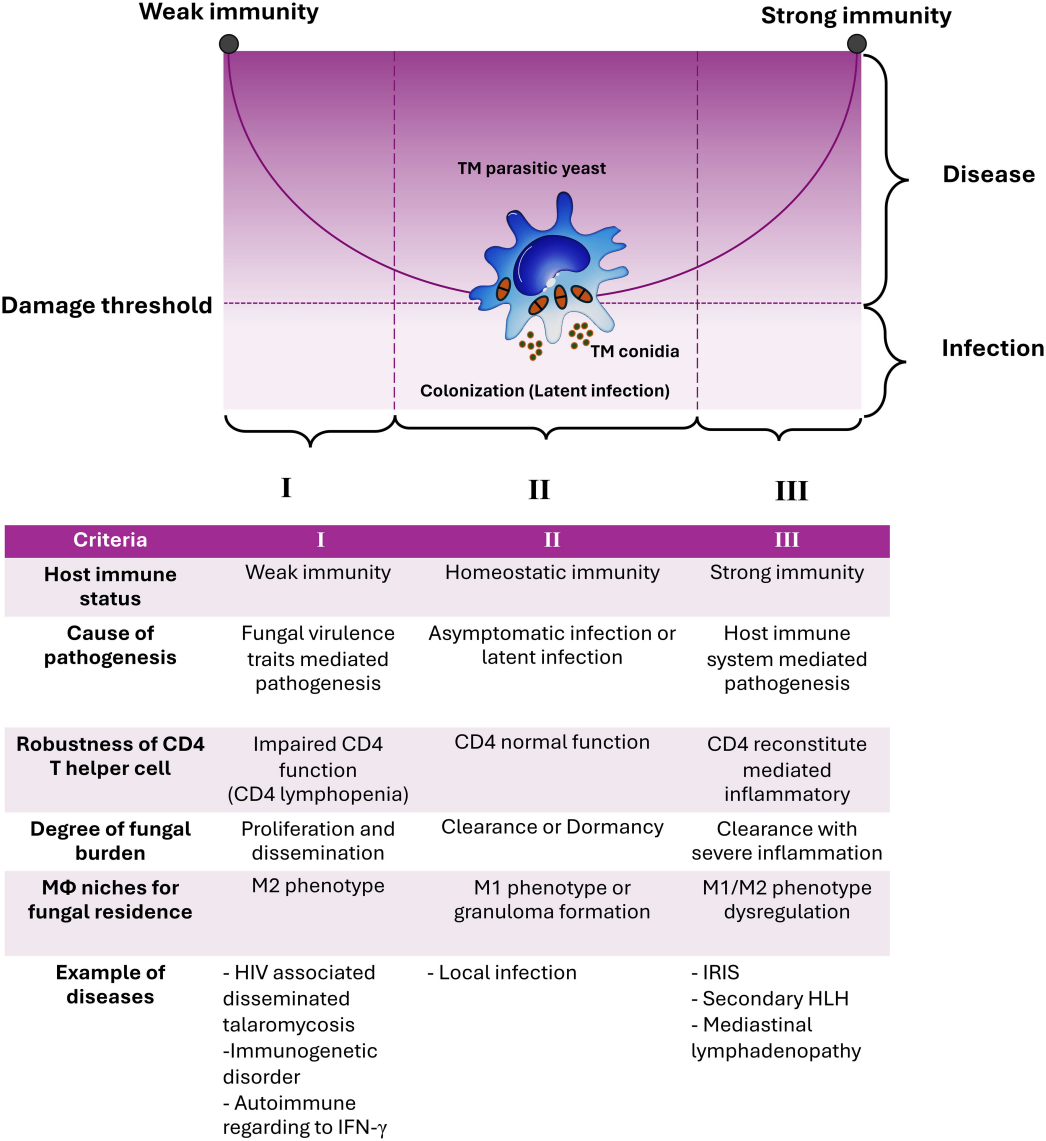
The damage-response framework (DRF) parabola and the pathogenesis of talaromycosis. The pathogenic yeast form of *T. marneffei* evades immune surveillance by transitioning from conidia to fission yeast parasitism within host macrophages (Mϕ). According to the DRF model, talaromycosis occurs only when significant damage occurs in the host due to the interaction between host factors and *T. marneffei* virulence traits. Criteria for such circumstances, including host immune status, cause of pathogenesis, robustness of CD4 T helper cells, degree of fungal burden, role of macrophage niches for fungal residence, and examples of diseases are shown. The DRF model describes the following host-microbe responses: Section I covers host-microbe interactions in hosts with weak immune responses, leading to disseminated or invasive disease, which can be life-threatening. Section II covers host-microbe interactions in hosts with normal immune responses that can contain or eradicate the infection, which is the most common scenario for human pathogenic fungi, thereby avoiding disease progression. Section III covers host-microbe interactions where the host mounts an excessively strong immune response, resulting in damage from overly aggressive inflammation, which can also be life-threatening. *T. marneffei* infections result in diseases occurring across the spectrum of host immune responses. This indicates that the host’s immune system directly influences pathogen elimination, disease progression, and disease severity. This figure has been modified from Casadevall and Pirofski, 2003 (15). (TM, *Talaromyces marneffei*; IRIS, Immune Reconstitution Inflammatory Syndrome; HLH, Hemophagocytic Lymphohistiocytosis).

The observations described above are readily apparent in the case of *T. marneffei* infection, which has been thoroughly documented and illustrated in various circumstances by comparing it to the graph of the conic sections of a parabola. The experimental and clinical evidence indicates that *T. marneffei* colonization may contribute to exaggerated inflammatory responses, representing host-mediated damage. Consequently, based on the current evidence, talaromycosis aligns with the class 4 of DRF, with *T. marneffei* acting as an “opportunistic fungus” and causing host damage in the presence of both strong and weak host responses. To our knowledge, this is the first formal application of the principles of DRF to explain the pathogenicity of *T. marneffei* infection (**Figure 1**).

## 3 *T. marneffei* attributes of virulence -related traits in mammalian host

Although the knowledge of *T. marneffei’s* lifestyle in the ecological (natural) environment remains limited, it can thrive independently, acting as a decomposer of organic matter. Similar to its kinsfolk within the genera *Penicillium* and *Talaromyces*, *T. marneffei* demonstrates a potential for beneficial application of its secondary metabolites, assuming it remains in its saprophytic mold form (16, 17). However, when environmental conditions shift, such as an increase in temperature to 37°C along with adequate nutritional factors (especially a rich nitrogenous source and acidifying pH), *T. marneffei* can transition from a saprophytic mold to a pathogenic yeast. Experimental evidence using human THP-1 macrophage cells indicates that this phase transition from conidia to yeast can occur within 12-24 hours (18). Conversely, in the absence of macrophage microenvironmental stress, *T. marneffei* conidia may take up to 2-3 weeks to transform into fully mature yeast forms in artificial medium. A critical question arises: what factors contribute to *T. marneffei* being the sole representative within the genera *Penicillium* and *Talaromyces*, as well as other close relatives within the phylum Ascomycota (order Eurotiales), exhibiting characteristics of thermal dimorphic fungi?

In general, the evolution of fungi thriving naturally and independently into animal pathogens, particularly endothermic animals such as mammals, poses significant challenges. *T. marneffei* can survive and proliferate within the cells of endothermic animals, such as humans, bamboo rats, dogs, and beech martens (*Martes foina*) (19). The cellular lifestyle of *T. marneffei* in natural reservoir animals (e.g. bamboo rats and dogs) is poorly understood, including whether it exists as intracellular fission yeast similar to what is observed in infected human tissues. Available data only suggests its presence within the mononuclear phagocytic system (MPS) involving organs, such as the liver and spleen of bamboo rats and beech martens. Thus, it is necessary to further elucidate the host and phase morphogenesis of *T. marneffei* in these known hosts as well as other potential mammalian hosts within endemic areas that could serve as reservoir hosts.

Phase transition from conidia to yeast of *T. marneffei* also occurs within free living amoeba. This is demonstrated with the amoeba *Acanthamoeba castellanii* at 37°C, where *T. marneffei* induces widespread cellular pathogenesis within the *A. castellanii* cells, leading to their destruction. Furthermore, the production of melanin by *T. marneffei* yeast within *A. castellanii* is linked to virulence (20), and this enigmatic pigment has been endorsed as a crucial virulence trait for the pathogenicity of nearly all fungal species (21, 22). However, further investigation into the interplay between *T. marneffei* and *A. castellanii*, as well as the underlying cellular and molecular mechanisms, is necessary. This study of *T. marneffei* and *A. castellanii* represents the first evidence demonstrating that mold to yeast phase transition of *T. marneffei* can occur within non-mammalian cells or hosts.

The characteristics of *T. marneffei* as a “soil organism” is particularly noteworthy as it is linked to mechanisms for host exposure to and acquisition of the fungus. Clear evidence supporting this notion includes the significant increase in outbreaks of this fungal species during the rainy season in endemic areas each year. This phenomenon is attributed to heavy rainfall disrupting soil and soil environmental systems, leading to the dispersal of *T. marneffei* conidia and promoting the growth of the saprophytic mold due to increased humidity (11). One hypothesis that could partly explain the origin of *T. marneffei* disease outbreaks is the amoeboid predator-animal virulence hypothesis (23). It suggests that fungi capable of causing disease in humans are more likely to encounter amoebae, which are resident micropredators in the soil, and selectively retain survival traits in these amoebae (such as conidia to yeast phase transition) to sustain these traits for future disease propagation in mammalian hosts. Nevertheless, this experimental design was conducted using an *in vitro* model. Further evidence is needed to confirm whether these events occur under natural environmental conditions. The hypothesis has been deeply explored with *Legionella pneumophila*, which co-evolved with amoebae and this bacterium exploits human macrophages during legionellosis (24). Intriguingly, environmental strains of the human commensal yeast *Candida albicans* can survive and adapt to withstand destruction by *A. castellanii*, whereas clinical isolates are eliminated by the amoeba (25). Moreover, the well-known virulence factors of pathogenic fungi, such as the melanized surface of fungal conidia and their ability to produce toxic secondary metabolites have also been found to protect *Aspergillus* spp. against the slime mold *Dictyostelium discoideum*. Recent advancements in studying interactions with human cells have led to the adaptation of other amoeba infection models in both fungivorous and non-fungivorous species. Both *D. discoideum* and *A. castellanii* have been extensively used as model organisms to study phagocytic interactions due to their similarity to human phagocytes in the innate immune system (26, 27). Based on these reasons, it can be inferred that amoeba predation and mammalian phagocytosis exhibit fundamental similarities in their methods of engulfing and processing prey or pathogens. Both systems rely on phagocytosis, a mechanism whereby cells internalize large particles that are then enclosed in a phagosome which merges with lysosomes to break down the ingested materials. This similarity in cellular processes means that virulence factors that evolved to resist amoebic digestion can also provide an advantage against mammalian phagocytes. For example, pathogens that develop strategies to avoid destruction by amoebae—such as producing enzymes that neutralize lysosomal contents or modifying surface proteins to escape detection—might similarly evade elimination by macrophages and neutrophils in mammals. Furthermore, pathogens that can survive and replicate within amoebae often exhibit analogous behavior in mammalian phagocytic cells, utilizing strategies evolved in amoebae to select their virulence traits in human hosts (28).

Survival of *T. marneffei* within macrophages involves a balance adjustment of the host cell through the M2 macrophage phenotype switch (or polarization) (29, 30), consequently inhibiting the fungicidal activity of M1 macrophages (31). Therefore, the behavior of *T. marneffei* yeast evading immune surveillance through the M2 macrophage phenotype appears to be a pivotal barrier preventing other immune cells and other immune mechanisms from destroying *T. marneffei* through several mechanisms, such as inhibiting JunB-mediated transcriptional activation of pro-inflammatory cytokines via the inhibition of histone acetylation (32). Additionally, protection of *T. marneffei* from destruction by neutrophil myeloperoxidase (33) and even the mechanism of “interleukocyte shuttling” (34) have been discussed.

Notably, the interaction between the M2 macrophage phenotype and pathogenic yeast of *T. marneffei* appears to be relevant to the Amoeboid Predator-Animal Virulence Hypothesis. The ability of *T. marneffei* to survive and proliferate within macrophage cells might derive from adaptations achieved through the environmental “bootcamp” of survival competition with amoebae or other predators that mimic macrophage biology (35–37). The proliferation of *T. marneffei* within M2 macrophages is a critical factor that enables its spread to other parts of the human body, likely initiating the right-hand side of the DRF parabola. This interaction also influences various T helper subsets. Extensive studies on *Cryptococcus neoformans* have shown that *C. neoformans* infection is facilitated by the stimulation of M1 macrophages and the subsequent induction of T_H_1 responses, which promotes intracellular clearance of the yeasts. In contrast, M2 macrophage activation is associated with T_H_2 immune responses, promoting the intracellular persistence of *C. neoformans* and contributing to the development of latency (38). Additionally, several studies have found that this immune escape strategy of *T. marneffei* could lead to stimulation of the right-hand side of the parabola curve of the DRF theory (10, 11, 39), ultimately leading to excessive inflammatory response and immunopathogenesis. These phenomena represent a dysregulation of the immune system, referred to as “the immune paradoxical reaction” (**Figure 1**).

## 4 Latency of talaromycosis: the lymphoid organ colonization and dormancy

Human resistance to *T. marneffei* is primarily mediated by a T_H_1 cellular immune response, which encompasses macrophage-mediated phagocytosis and delayed hypersensitivity facilitated by sensitized T cells, the latter response plays an important role in latent infection. Latency or dormancy in *T. marneffei* infection is well documented, particularly in instances involving tourists visiting *T. marneffei* endemic regions who develop talaromycosis long after returning to their home countries. Latent *T. marneffei* infections are believed to contribute to the increased severity of infection and a tendency for recurrence. For example, among 14 individuals with HIV who developed talaromycosis after travel to a *T. marneffei* endemic region, 10 exhibited *T. marneffei*-related symptoms within 12 months, while another patient developed bloodstream infection 18 months post-monitoring. Thus, during the early stages of HIV infection when the host immune system remains largely intact, *T. marneffei* may remain asymptomatic, potentially accompanied by a period of fungal latency. As HIV progresses and the robustness of the immune system declines, or in cases of immunosuppression, latent infections may evolve into disseminated *T. marneffei* infections. These occurrences suggest that *T. marneffei* may utilize diverse strategies to evade host immunity, facilitating latency/dormancy (40).

Human *T. marneffei* acquisition occurs after inhalation of conidia or hyphal fragments and their deposition in the lungs (41). Infection requires evasion of various host receptors of alveolar tissues (11). The shift from saprophytic to yeast from further complicates the host response as different antigens are presented by the fungus (42). Recent reports also indicate that human bronchial epithelial cells can act as reservoirs for *T. marneffei*, with intracellular yeast hiding within these cells, making them immunological privileged sites for the fungus to evade the immunosurveillance activity (43). Additionally, regulating the expression of programmed death-ligand 1 (PD-L1) or CD274 by stellate macrophages (Kupffer cells) is noted as another strategy for *T. marneffei* to circumvent immune recognition (40).

Several intracellular pathogens, such as *Listeria monocytogenes*, *Mycobacterium tuberculosis*, *Toxoplasma gondii* and *Trypanosoma cruzi*, can induce macrophage polarization toward the M2 (44). This M1/M2 polarization influences their ability to eliminate these intracellular pathogens versus allowing the microbes to persist or proliferate (44, 45). In healthy individuals, macrophages may eliminate *T. marneffei*, but they can also act as reservoirs for *T. marneffei* persistence or proliferation and may also play a role in T_H_1 impairment. Data from an *in vitro* THP-1 human macrophage model shows that *T. marneffei* and its immunogenic derivatives from extracellular vesicles have developed mechanisms to evade killing by M1-activated macrophages and the fungus promotes a phenotypic shift to the M2 (29, 46, 47), thereby facilitating latent or dormant infection. M1 macrophages are essential during the initial phases of fungal infection and granuloma formation by providing a strong inflammatory response and actively engulfing pathogens. Following this, the enzyme inducible nitric oxide synthase (iNOS) derived from L-arginine plays a crucial role in producing nitric oxide or its derivatives to further kill the pathogens. Therefore, iNOS production is recognized as a hallmark of M1 macrophage polarization (48). In studies involving *Coccidioides* sp., iNOS is likely involved in the macrophage phagocytosis mechanism in coccidioidomycosis. The absence of iNOS probably leads to a reduced ability to effectively phagocytose *Coccidioides* sp. or form and maintain effective granulomas. Although iNOS from M1 macrophages may not increase host survival, it contributes to the maintenance of granulomas and restricts dissemination. Therefore, M1 macrophages seem to regulate granulomas even though it may not completely eradicate the intracellular pathogen (49). When discussing the latency or dormancy of talaromycosis, the mechanism of macrophage polarization in *T. marneffei* infection plays a crucial role in such conditions. If the M2 polarization of the macrophage paradox effect serves as a favorable niche for host immune evasion, it implies that M2 macrophages can act as “Trojan horses,” transporting parasitic *T. marneffei* throughout the body. This so-called “spreading *via* transmigration of infected phagocytes” (50) leads to disseminated talaromycosis and the “Trojan horse” mechanism may also potentially breach the blood-brain barrier to enter the central nervous system (51), which is a process experimentally well-characterized in cryptococcosis (52).

**Figure 2:**
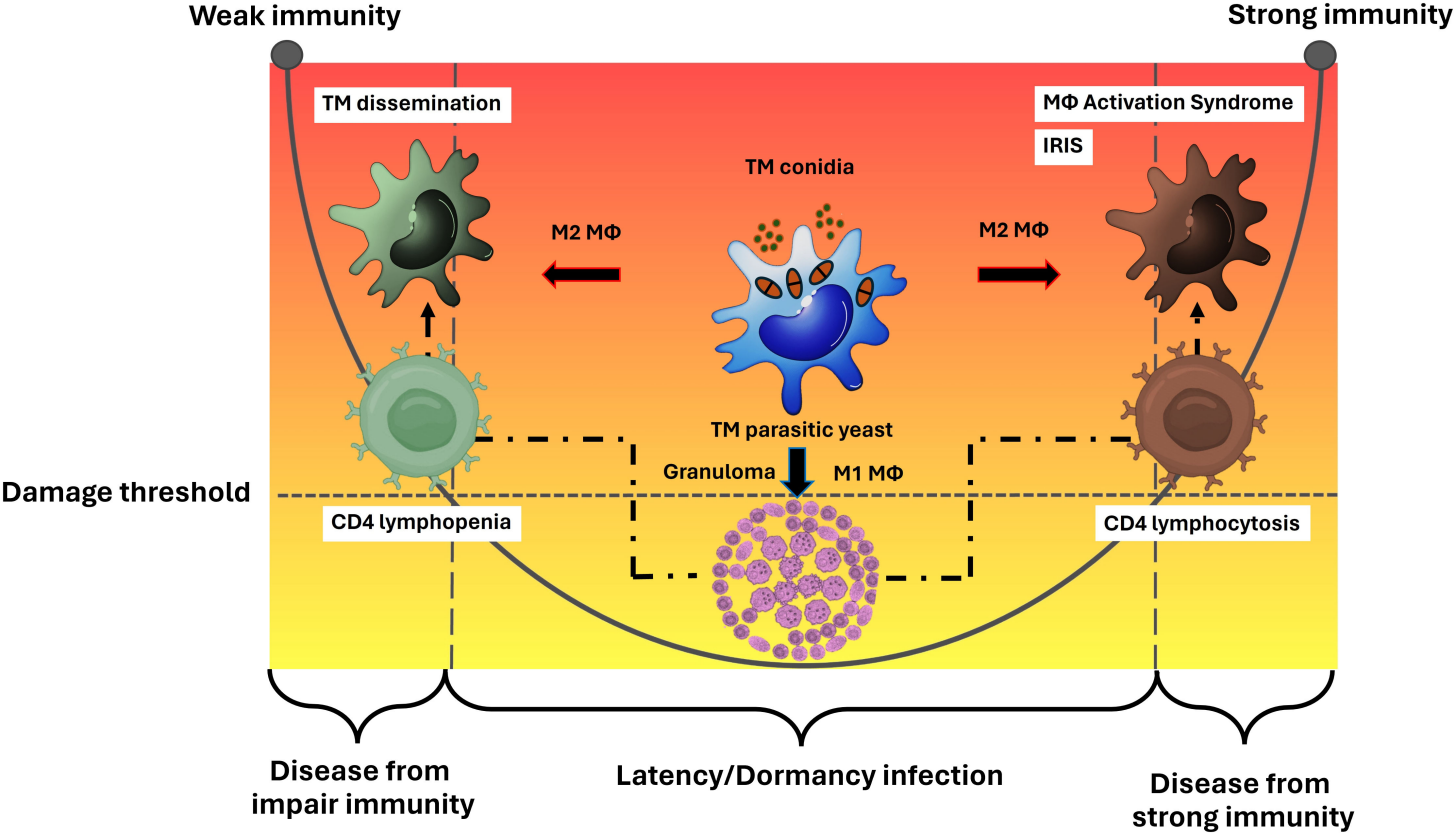
Macrophage effector functions versus macrophage paradoxical functions in the immunopathogenesis of talaromycosis and the DRF parabola. Macrophage (Mϕ) polarization is a crucial factor in either eliminating or permitting the survival of *T. marneffei* within the host. The M1 phenotype typically leads to the eradication of the pathogen, but in some cases where the immune system is compromised, particularly in the context of a deficient cell mediated immune response, M1 macrophages may fail to eliminate the pathogen. M1 macrophages often resist infection by forming granulomas, which prevent the spread of *T. marneffei*, leading to latent or dormant talaromycosis. This process involves CD4 T helper cells. When CD4 levels decrease, *T. marneffei* can disseminate throughout the human body via the mononuclear phagocytic system, leading to diseases depicted on the left side of the parabolic curve. Conversely, a sudden increase in CD4 levels after a period of depletion (such as in the treatment of HIV infection) can cause severe inflammatory disease, as observed in talaromycosis-associated immune reactivation inflammatory disease (IRIS), represented on the right side of the parabolic curve. On the other hand, the M2 phenotype allows *T. marneffei* to survive and M2 macrophages act as vehicles for spreading the infection throughout the human body leading to systemic or disseminate talaromycosis. This mechanism is an importance tactic for *T. marneffei* yeast cells to escape the host immune system, resulting in diseases on the left side of the parabolic curve. Similar to other severe infections from intracellular pathogens, like cryptococcosis or tuberculosis, M2 macrophages can lead to dysregulated macrophage activation, resulting in a severe cytokine storm known as Macrophage Activation Syndrome (MAS) or secondary Hemophagocytic Lymphohistiocytosis (HLH), which also leads to diseases on the right side of the parabolic curve. Therefore, a deeper understanding of the function and dysfunction (paradox) of host macrophages infected with *T. marneffei* are necessary for clearly elucidating the immunopathogenesis of talaromycosis. This figure has been modified from Casadevall and Pirofski, 2003 (15). (TM, *Talaromyces marneffei*).

Overall, the M2 macrophage phenotype facilitates the survival of pathogenic *T. marneffei* and serves as a means for disseminating the infection throughout the human body, ultimately leading to a systemic or disseminated infection. This strategy is a key method for *T. marneffei* yeast cells to evade the host immune system, resulting in diseases associated with the left side of the DRF parabolic curve. Similarly, in severe infections caused by intracellular pathogens, like cryptococcosis or tuberculosis, M2 macrophages can cause uncontrolled macrophage activation, which may lead to a severe cytokine storm known as Macrophage Activation Syndrome (MAS) or secondary Hemophagocytic Lymphohistiocytosis (HLH), reflecting the disease outcomes on the right side of the parabolic curve. Thus, a comprehensive understanding of both the functional and dysfunctional roles of macrophages infected with *T. marneffei* is crucial for accurately defining the immunopathogenesis of talaromycosis (**Figure 2**).

In contrast to the relatively permissive dissemination afforded by M2 macrophage harboring virulent *T. marneffei*, granuloma formation is a protective response to infection with this fungus. The formation of a granuloma involves collaboration between M1 macrophages and the acquired immune system to control infection. Granulomas are observed in the lungs of *T. marneffei* infected mice (53) and in tissues from patients with talaromycosis (54). The formation of granulomas to control fungal infection is orchestrated by IFN-γ, tumor necrosis factor (TNF), and IL-6 from T_H_1 cells, along with partial support from T_H_17 cells, which collaborate with other immune cells to cooperatively control *T. marneffei* infection and prevent systemic infection (55).

The role of granulomas in *T. marneffei* infection is complex and the overall host immune milieu influences whether the process can control the disease. For example, there are three histological categories of hepatic human talaromycosis: diffuse, granulomatous, and mixed patterns (54). Notably, *T. marneffei* infection in non-hepatic human tissues can manifest in suppuration and granuloma formation with necrosis lesions especially, in immunocompromised individuals. Experimental investigations with *T. marneffei* demonstrate that mice can acquire infection through either respiratory or parenteral routes, often involving hepatic complications. Immunocompetent mice typically mount a granulomatous response to the pathogen, ultimately clearing the infection. However, congenitally immunodeficient strains of mice initially develop granulomas, but progress to systemic disease with livers characterized by parenchymal replacement by *T. marneffei* and proliferation of macrophage containing yeast cells. These murine experiments demonstrate the critical role of an effective cell mediated immune response in defense against this fungus (53, 56). Nevertheless, it should be noted that while the main role of granuloma is to contain the fungal infection, these lesions paradoxically also provide a protective environment, enhancing pathogen latency (57).

From another perspective, the conditions of latency/dormancy and granuloma formation could be particularly advantageous when considering the prevention of talaromycosis through active surveillance policy, especially in endemic areas. Current concepts include reports on preliminary establishing IFN-γ release assays for latent talaromycosis (IGRA) to detect sensitized T lymphocytes using the Mp1p recombinant antigen, which is an intriguing topic that requires further investigation (58). This particularly focuses on the specificity and sources of lymphocytes producing IFN-γ, which links to the possibility of stimulation through bystander-activated CD8+ T lymphocytes, along with host-based assays or other surrogate host-based assays beyond the conventional antigen-antibody system specific to *T. marneffei* infection. The traditional antigen-antibody assays typically produce positive outcomes for *T. marneffei* infection solely in instances of widespread dissemination or systemic infection. This approach may not allow the clinician to immediately provide appropriate management to a patient with talaromycosis.

## 5 Disseminated talaromycosis in immunodeficiency: the left-hand side of the parabola

Healthy individuals with competent immune systems are generally capable of eliminating *T. marneffei*. In contrast, those with compromised immune systems are significantly more susceptible to both active and latent infections, which are well characterized in individuals with advanced HIV. However, *T. marneffei* infection can also occur in other conditions of impaired immunity, primarily those with a congenital immunodeficiency (immunogenetic disorder) (59, 60). Disseminated talaromycosis resulting from immunodeficiency represents the quintessential manifestation of this disease, primarily attributable to abnormalities in cell mediated immune responses, particularly in a deficient T_H_1 response. For instance, HIV infection leading to CD4 levels below 50-100 cells/µL, along with various dysfunctions associated with IFN-γ, create a condition permissive for disseminated talaromycosis. Current evidence strongly supports the direct correlation between impaired T lymphocytes (CD4 and CD8) and the underlying causes of dysregulation in IFN-γ release and IFN-γ intracellular signaling cascades (61, 62). The literature on talaromycosis often categorizes the etiology of *T. marneffei* infection based on the presence or absence of HIV infection (HIV/AIDS vs non-HIV/AIDS talaromycosis). The predominantly described non-HIV/AIDS talaromycosis cases are attributed to genetic abnormalities related to the immune system and categorized as primary immunodeficiency syndromes. These include defects in the IFN-γ/STAT1 signaling pathway (including gain-of-function STAT1 related diseases), elevated levels of serum anti-IFN-γ neutralizing autoantibodies (or adult-onset immunodeficiency syndrome; AOID), STAT3 Hyper IgE syndrome, mutations in the CD40 ligand (CD40L or CD154) gene, X-linked hyper-IgM syndrome, malignancy and targeted therapy for anti-cancer (11). These various aberrant conditions invariably predispose individuals to disseminated *T. marneffei* infection.

As detailed above, intact cellular immunity is required, particularly T_H_1 and M1 polarization of macrophages, for control of *T. marneffei.* Interestingly, macrophages in *Danio rerio* (zebrafish) embryos can shield *T. marneffei* from neutrophil-mediated cytotoxicity through myeloperoxidase and related events known as fungicidal neutrophil extracellular traps (NETs), emphasizing macrophage niche as a protective mechanism in *T. marneffei* latency (33). This is similar to research using larval zebrafish models infected with the pathogenic *Aspergillus fumigatus*, which observed macrophages capturing *A. fumigatus* spores. These macrophages aid the fungus in surviving by inhibiting spore germination and protecting them from being killed by polymorphonuclear neutrophils. Specifically, macrophages cluster tightly around the injected spores. When spore germination is suppressed, the recruitment and killing activity of neutrophils decrease. Consequently, macrophages act as protective niches for *A. fumigatus* spores against neutrophils-mediated killing, thereby facilitating lung colonization (63). This aspect is correlated with the impaired functionality of T_H_17, as the priming of helper T cells by dendritic cells (as well as other professional antigen presenting cells) inhibits the stimulation of the T_H_17 pathway, which plays a crucial role in regulating the recruitment of neutrophils to sites of infection (64, 65). The role of T_H_17 in *T. marneffei* infection warrants further investigation, particularly concerning its involvement in acquired immunity. However, T_H_17 is known to play a significant role alongside T_H_1 in controlling innate NLRP-3 inflammasome through caspase-1-dependent release of pro-inflammatory cytokines (such as IL-1β), as well as the pyroptosis triggered by *T. marneffei* yeast cell components (66).

## 6 Disseminated talaromycosis from paradoxical reactions or other excessive immune activations: the right-hand side of the parabola

Inflammatory responses during infection are a double-edged process as the host effector activities can lead to the eradication of invading microbe, but the activation of immune cells and their release of inflammatory molecules can directly and indirectly damage diverse host cells and tissues, sometimes severely. Systemic inflammation significantly influences the overall symptoms of disseminated talaromycosis, not only during its initial stages but also upon commencement of effective antimicrobial treatment or during immune reactivation. This phenomenon is exemplified by the “immune paradoxical reaction” characterized by the worsening of pre-existing lesions or the appearance of new ones during antifungal therapy and with the administration of antivirals to individuals with HIV disease. This condition is considered a life-threateningly exaggerated or dysregulated inflammatory syndrome, which represents a severe complication of talaromycosis. There are two reported forms of this syndrome in talaromycosis: Immune Reconstitution Inflammatory Syndromes (IRIS) and macrophage activation syndrome (MAS) or secondary (acquired) hemophagocytic lymphohistiocytosis (HLH). The prominent disease mediator in IRIS is the T helper cell (67), whereas macrophages are the key players in MAS/HLH (68).

Talaromycosis-associated IRIS is frequently encountered in patients co-infected with HIV in whom antiretroviral therapy is initiated, significantly boosting the CD4 T cell levels within a short period of time. IRIS manifestation can be further subdivided into two categories: the uncovering of previously “occult” or subclinical infections (unmasking IRIS) and a “paradoxical” worsening of a treated or under treatment infection (paradoxical IRIS). Numerous reports have documented talaromycosis-associated unmasking IRIS, while talaromycosis-associated paradoxical IRIS has become increasingly well recognized (69). Although talaromycosis-associated unmasking and paradoxical IRIS occur at different stages post-diagnosis, their immunopathogenesis may share similarities. There are some notable observations, in patients with disseminated talaromycosis, paradoxical IRIS is reported less frequently than unmasking IRIS and the origin of pathogenesis remains unclear and requires further intensive study. However, the clinical manifestations between two types of the IRIS differ, with paradoxical IRIS having a longer onset period of more than 12–20 weeks (1–8 weeks for unmasking IRIS). Additionally, the skin lesions observed in paradoxical IRIS patients tend to exhibit severe inflammation and atypical features, similar to those found in conditions like erythema nodosum and psoriasis, and also exhibit the pathological presence of comma vessels (69, 70). Not solely due to T lymphocyte dysregulation, hyper-responsive innate phagocytic cells, which lead to excessive inflammation upon T cell reconstitution (due to retrovirus reduction), have been identified as culprits of IRIS. Moreover, in tuberculosis-associated paradoxical IRIS, substantial evidence now shows that monocyte-macrophage (CD14^++^CD16^-^) activation is a key factor in IRIS immunopathogenesis (71). Like other fields of talaromycosis knowledge, there is still a significant gap in explaining the elucidated immunopathogenesis of these abnormal conditions, necessitating further study.

Notably, there have been no systematic clinical or immunological studies of talaromycosis-associated IRIS. In fact, in the setting of HIV co-infection, an IRIS event is believed to arise from an immune response targeting an active, often latent, infection caused by an opportunistic pathogen or antigens from non-viable pathogens or their residues, especially intracellular pathogens. Generally, IRIS is driven by both the host immune responses and the pathogen load. The primary risk factors for IRIS include lower CD4 T cell counts, inadequate CD4 T cell recovery following ART regimen, an active or subclinical opportunistic infection with a high pathogen burden from latent/dormant stages, and elevated frequencies of antigen-specific T cells poised for activation once immunodeficiency state is rescued (67). Examples of fungal infection-associated IRIS are well documented in the context of cryptococcosis. The excessive inflammation in the central nervous system caused by cryptococcal meningitis-associated IRIS can be life-threatening. Unlike unmasking disease, paradoxical cryptococcal IRIS arises after the diagnosis and treatment of the infection and the commencement of antiretroviral therapy (ART). This type of IRIS manifests anywhere from a few days to several years after starting ART in patients with cryptococcal meningitis. Although unmasking cryptococcal IRIS and paradoxical cryptococcal IRIS occur at different stages relative to diagnosis, their underlying mechanisms may share similarities (72). Therefore, a careful assessment for underlying latent infections is crucial before starting antiretrovirals in individuals with HIV/AIDS. For example, targeted screening for cryptococcosis is recommended prior to antiretroviral initiation (73). However, there is currently no commercial test used for screening patients for talaromycosis. Host-based assays, such as IGRA, are likely to be essential tools for managing talaromycosis alongside ART regimens to avoid talaromycosis-associated IRIS.

HLH is characterized by macrophage cells excessively engulfing various hematological cells, such as erythrocytes, other leukocytes (myeloid series), and platelets. Macrophage-mediated HLH has a strong association with the onset of MAS in individuals infected with intracellular microorganisms, including *T. marneffei*. As a result, a direct connection has been established between impaired perforin (from NK cells and cytotoxic CD8 T lymphocytes) mediated cell lysis and the subsequent hypercytokinemia or atypical cytokine storm associated with HLH. Many studies have suggested that hemophagocytic macrophages have an M2 phenotype, however, the macrophage phenotype exists as a spectrum, partly attributable to the plasticity or heterogeneity of the macrophages (68). The etiology of *T. marneffei* infection associated with HLH may encompass a severe inflammatory response syndrome arising from congenital immunogenetic defects (in cases of non-HIV associated talaromycosis), post-infection immune deficiencies, or severe infection. Dysregulation of immunomodulatory processes, accumulation of immunocompetent cells, and heightened production of pro-inflammatory cytokines are pivotal factors contributing to HLH pathogenesis. Animal models demonstrate significant contributions of CD8 T lymphocytes and NK cells in IFN-γ production to the pathobiology of HLH. CD4 T cells are implicated in mediating immunodeficiency, particularly in the setting of HIV. Notably, abnormalities in CD4 T cells, CD8 T cells, and immunoglobulin levels are common in HLH, which gradually normalized as disease status improves, suggesting a potential for *T. marneffei* infection to manipulate immune dysregulation. Interestingly, there is a higher prevalence of HLH in non-HIV associated pediatric talaromycosis compared to HIV-associated talaromycosis. Nonetheless, the mechanisms and diagnosis of adult- and pediatric- onset HLH remain poorly elucidated, necessitating further research (74, 75).

The roles of CD8 and dendritic cells in *T. marneffei* infection have not been critically assessed. This area may be important as these cells are important in the pathogenesis of the thermally dimorphic fungus *Histoplasma capsulatum* where interactions between dendritic cells and CD8 can present intracellular exogenous *H. capsulatum* antigens (antigens not originating from the dendritic cell itself) through the process of cross-presentation (cross-priming) via MHC class I molecules. Specifically, Histoplasma-specific CD8 T cells are primed by dendritic cells presenting exogenous *Histoplasma* antigens, which occurs either through direct phagocytosis of the *Histoplasma* yeast or by uptake of fungal antigen remnants associated with the apoptotic bodies of infected macrophages (76).

## 7 The gap in T_H_2 response knowledge in the setting of talaromycosis

There are many gaps in our knowledge on *T. marneffei* pathobiology. There is, for example, limited information regarding the involvement of humoral immune responses (HIR), such as the complement system and antibody response, in talaromycosis. Due to the compromised immune state commonly observed in individuals with talaromycosis, significant deficiencies in humoral (antibody-mediated) immune responses are likely. Although antibody responses are frequently thought to play a limited role in eliminating intracellular pathogens, antibodies can protect in diseases with intracellular yeast, such as *H. capsulatum* and *C. neoformans* (77). Consequently, comprehensive investigations into this aspect of host immunity are required to elucidate the role antibody-mediated responses in talaromycosis. *T. marneffei* antigens, such as cell wall mannoproteins, are capable of stimulating robust antibody responses. Using a highly immunogenic secreted cell wall mannoproteins, Mp1p, mice immunized with the recombinant protein and DNA generated specific IgM and IgG antibodies (78). However, the impact of these antibodies on disease progression remains unknown. A large amount of data concerning humoral immunity to *T. marneffei* have been obtained through serological assays for clinical laboratory diagnosis, identifying strongly immunoreactive proteins or mannoproteins, such as crude cytoplasmic yeast antigen (*T. marneffei* CYA), Mp1p, Mplp6, and HSP30, based on their recognition by serum-specific antibodies in individuals with talaromycosis (79, 80). Although immunocompetent mice generated robust responses to Mp1P, patients co-infected with *T. marneffei* and HIV exhibit low or undetectable levels of antibodies to the mannoproteins. Additionally, certain other *T. marneffei* proteins considered as highly immunogenic also fail to elicit antibody production in immunocompromised patients (10). However, there nevertheless may be a crucial interplay between humoral and cellular immunity relevant to talaromycosis, as protective antibodies are necessary for robust CD4 T cell- promoted immunoglobulin class switching (isotype switching) and somatic hypermutation (affinity maturation). In the context of immunodeficiency, particularly in the setting of AIDS and T-cell immunodeficiencies, disruptions in B cell function hinder the efficient generation of high-affinity antibody responses against HIV and other opportunistic pathogens. Consequently, while antibody production may theoretically offer initial protection, without further reinforcement through interactions with CD4 T cells, sustained functional antibodies for prolonged protection are absent. Notably, such a defect in antibody production has been observed in other AIDS-associated systemic mycoses caused by dimorphic fungi, including histoplasmosis and blastomycosis (10).

An alternative viewpoint derived from previous investigations conducted by our research team highlights the considerable challenge of identifying protective or hallmark antibodies highly specific to *T. marneffei*. This challenge arises due to the highly conserved nature of immune responses to fungal antigens across various fungal pathogens (10). Moreover, the presence of shared antigens between saprophytic molds and pathogenic yeast renders the differentiation of antibodies from these two phases unfeasible with current technology. For *Paracoccidioides brasiliensis*, *H. capsulatum*, and *Blastomyces dermatitidis*, identifiable immunogens associated with protective immunity exist, such as gp43 for *P. brasiliensis*, the H antigen or M antigen for *H. capsulatum*, and Blastomyces adhesin 1 (BAD 1) for *B. dermatitidis* (81). A hallmark protective antibody against *T. marneffei* has not been identified, primarily because the antigen pivotal to cause of the pathogenesis of this fungus remains elusive. Our screening endeavors have been limited to identifying candidate antigenic protein through inference from other fungal pathogens (10, 82). Presently, only the Mp1p protein stands out as the most prominent and extensively characterized antigen. However, further investigations are required to elucidate the precise role of this protein in *T. marneffei* pathogenesis (83–85). Hence, the knowledge gap of T_H_2 impairs our capacity to fit this arm of the immune system into the DRF for *T. marneffei* infection.

## 8 Conclusion and future perspectives

At this moment, this is what we aim to emphasize, our focus lies in highlighting the process of dissecting the steps associated with *T. marneffei* intracellular parasitism establishment and its intricate interactions with the diverse array of the immune system, as influenced by the host DRF. This will significantly advance our comprehension of *T. marneffei* pathogenesis. The extensive array of immunological mechanisms contributing to the pathology of fungal infections cannot be captured within a single narrative review. Three decades ago, much of the research in this field was centered around investigating the roles of immune complexes, cytokines, and the behavior of fungi within host macrophages. While these areas remain pertinent, there has been a shift in focus towards elucidating the molecular fundamentals of cellular processes such as overly aggressive inflammation, granuloma formation, and local tissue damage. A pivotal concern is understanding how the host maintains a delicate balance between mounting a protective immune response and one that predisposes to pathological complications. It is increasingly evident that this balance represents a critical determinant of the success of the host- *T. marneffei* relationship.

Moreover, after looking the pathogenesis of *T. marneffei* through the DRF framework, several questions remain to be thoroughly explored, including which human host receptors are involved in the recognition of *T. marneffei* saprophytic conidia versus its pathogenic yeast form and are they the same or different? What protective immune signaling pathways are activated? Which key virulence traits directly modulate host immune responses? What cellular and molecular mechanisms are associated with *T. marneffei*-macrophage interactions? Another intriguing area for future research is the potential long-term impact of these interactions on the subsequent dysregulation of macrophages by secondary HLH. The interactions between host macrophages and the fungus may contribute to the development of innate immune memory, which can result in either a manipulated immune response (latency/dormancy) or enhanced innate immune memory (trained immunity). Future studies in this field may also reveal that fungal infection promotes trained immunity in pulmonary alveolar macrophages and epithelial cells, enhancing the first line of barrier immunity against *T. marneffei* infection (86, 87).

Finally, the understanding of polarization, function, and interaction of macrophages with *T. marneffei* is essential for controlling the progress of infections and developing future immunotherapies as well as the active immunization, as they are the first line of defense in the innate immune response against facultative intracellular pathogens like *T. marneffei*. Identifying specific fungal factors that interact with host macrophages is crucial for these efforts. These pathogenic capabilities not only enable their survival trajectory but also allow them to access other tissues by transmigrating through infected macrophages. During this process, *T. marneffei* induces macrophages to perform a dual role-either clearing the infection or proliferating, which can either control the talaromycosis or facilitate spread the infection. This pathogenic mechanism, encompassing a range of strategies, enhances the virulence of this thermally dimorphic fungus.
